# A Pilot Study of Hepatic Irradiation with Yttrium-90 Microspheres Followed by Immunotherapy with Ipilimumab and Nivolumab for Metastatic Uveal Melanoma

**DOI:** 10.1089/cbr.2021.0366

**Published:** 2022-02-08

**Authors:** David R. Minor, Kevin B. Kim, Ricky T. Tong, Max C. Wu, Mohammed Kashani-Sabet, Marlana Orloff, David J. Eschelman, Carlin F. Gonsalves, Robert D. Adamo, Pramila R. Anne, Jason J. Luke, Devron Char, Takami Sato

**Affiliations:** ^1^ Center for Melanoma Research and Treatment, California Pacific Medical Center Research Institute, San Francisco, California, USA.; ^2^ Departments of Medicine, Radiology, and Ophthalmology, California Pacific Medical Center, San Francisco, California, USA.; ^3^ Departments of Medicine and Radiology, Thomas Jefferson Medical School, Philadelphia, Pennsylvania, USA.; ^4^ Department of Medicine, University of Chicago, Chicago, Illinois, USA.

**Keywords:** uveal melanoma, ipilimumab, nivolumab, Yttrium-90, hepatic metastases

## Abstract

***Background:*** Liver metastases from uveal melanoma carry a very poor prognosis. Hepatic artery infusions with Yttrium-90 (^90^Y) resin microspheres have some activity in this disease, and radiation and immunotherapy may be synergistic. The primary objective of this study was to determine the safety and tolerability of sequential ^90^Y resin microspheres and immunotherapy with ipilimumab and nivolumab in metastatic uveal melanoma.

***Materials and Methods:*** Twenty-six patients with uveal melanoma with hepatic metastases were entered into a pilot study. Treatment consisted of two infusions of ^90^Y resin microspheres, one to each lobe of the liver, followed in 2–4 weeks by immunotherapy with ipilimumab and nivolumab every 3 weeks for four doses, then maintenance immunotherapy with nivolumab alone.

***Results:*** Initial dosing of both ^90^Y and immunotherapy resulted in excessive toxicity. With decreasing the dosage of ^90^Y to limit the normal liver dose to 35Gy and lowering the ipilimumab dose to 1 mg/kg, the toxicity was tolerable, with no apparent change in efficacy. There was one complete and four confirmed partial responses, for an objective response rate of 20% and a disease control rate of 68%. The median progression-free survival was 5.5 months (95% confidence interval [CI]: 1.3–9.7 months), with a median overall survival of 15 months (95% CI: 9.7–20.1 months).

***Conclusions:*** With dose reductions, sequential therapy with ^90^Y and immunotherapy with ipilimumab and nivolumab is safe and tolerable, and has activity in metastatic uveal melanoma. These results justify a controlled trial to demonstrate whether ^90^Y resin microspheres add to the utility of combination immunotherapy in this disease.

Clinical Trial Registration number: NCT02913417.

## Introduction

Uveal (eye) melanoma is a rare cancer with about two thousand cases per year in the United States.^1^ Despite advances in diagnosis and local tumor control, the overall mortality rate of uveal melanoma remains high, with a 5-year survival of ∼40%, because of the development of metastatic disease, often many years after treatment of the primary cancer. Over 90% of patients with metastatic disease have liver involvement.^2^ The survival of patients with metastatic uveal melanoma remains poor, with a median survival of ∼6–12 months.^3,4^ Long-term survival from metastatic uveal melanoma is almost unknown, and there is no treatment that the Food and Drug Administration (FDA) has approved specifically for uveal melanoma.

In contrast to cutaneous melanoma, mutations in *BRAF* are very rare in uveal melanoma, whereas mutations in *GNAQ* and *GNA11* are common,^5^ and uveal melanoma has a much lower tumor mutational burden. Uveal melanoma incidence does not appear related to ultraviolet light exposure, and the reasons for the predilection for hepatic metastases remains unknown. Because of the *GNAQ*/*GNA11* mutations it was hoped that therapy with agents targeting MEK would be helpful, but trials with selumetinib^6^ and trametinib were negative.

Perhaps because of the low tumor mutational burden, single agent trials of checkpoint inhibitors ipilimumab,^7^ tremilimumab, nivolumab, and pembrolizumab^8^ have been disappointing, with response rates of 5% or less. At the time this trial was started, there was no significant published data on combination checkpoint inhibitor use for uveal melanoma. However, we hypothesized that the combination of ipilimumab and nivolumab would be superior to their use as single agents, although with less efficacy than that observed for cutaneous melanoma.

A variety of local therapies have been used for hepatic metastases including surgery, radiofrequency ablation, percutaneous hepatic perfusion with melphalan, immunoembolization, chemoembolization, radioembolization, and external beam irradiation. No local modality has shown a proven survival benefit. Recently, we have studied Yttrium-90 (^90^Y) resin microspheres to deliver hepatic internal radiation with safety and some evidence of efficacy.^9,10^ Because of the literature suggesting a synergistic effect when radiation and immunotherapy are combined,^11,12^ we felt a pilot study of hepatic internal radiation with ^90^Y resin microspheres followed by immunotherapy would be useful.

## Materials and Methods

### Study design and participants

This prospective pilot clinical trial was conducted at three centers with prior experience with both ^90^Y resin microspheres and immunotherapy. Patients were accrued between March 2017 and February 2021. Adult patients were eligible if they had uveal melanoma with hepatic metastases, disease measurable by iRECIST,^13^ performance status of 0 or 1, and less than 50% of the liver involved. The study began with the FDA-approved doses of both ^90^Y resin microspheres and combination immunotherapy, with close monitoring of toxicity by the principal investigators and data safety monitoring board (DSMB). Protocol design included specific dose-limiting toxicities and the requirement for holding accrual after six patients to evaluate toxicity.

The study was approved by the FDA under an Investigational Device Exemption number G150186 issued October 6, 2016, and the FDA approved all protocol amendments. The trial was registered at Clinicaltrials.gov. The trial was sponsored by the California Pacific Medical Center Research Institute and data analysis was performed there by a physician unrelated to the principal investigators at each site. The protocol, consent forms, and amendments were approved by the Human Subjects Institutional Review Boards of Sutter Health (California Pacific), Thomas Jefferson University, and the University of Chicago.

### Procedures

Before any treatment, patients had routine laboratory tests and imaging of the liver with MRI scans, except for a single patient with a pacemaker who had CT scans throughout. Patients then underwent a hepatic angiogram with mapping of the hepatic arterial system, embolization of ectopic vessels, and calculation of possible pulmonary shunting of radioisotope through a technectium-99m (^99m^Tc) macro-aggregated albumin scan obtained after injection of ^99m^Tc into the hepatic artery. Patients were then treated with two doses of ^90^Y resin microspheres through the hepatic artery, one to each lobe of the liver, 2 to 4 weeks apart. Dosage calculation for the ^90^Y initially was done by the “BSA” method described in the package insert,^14^ but was subsequently reduced when excessive toxicity was seen.

Immunotherapy was initiated 3 to 4 weeks after the second dose of ^90^Y provided liver function tests were at the grade 0–1 toxicity level. Initial dosing of immunotherapy was ipilimumab 3 mg/kg and nivolumab 1 mg/kg every 3 weeks for four doses, followed by nivolumab 3 mg/kg every 2 weeks for up to 3 years. Due to excessive toxicity, after the first eight patients, the DSMB decided to limit the ^90^Y dose to the normal liver to 35 Gy and reduce the ipilimumab dose to 1 mg/kg. When those doses proved tolerable in the next nine patients, the dose of nivolumab during the initial immunotherapy phase was increased to 3 mg/kg as the nivolumab 3 mg and ipilimumab 1 mg regimen was becoming increasingly used for cutaneous melanoma.

Thus, patients were treated at three distinct dose levels: package insert dose of ^90^Y with nivolumab 1 and ipilimumab 3 mg/kg, and reduced dose yttrium with ipilimumab 1 mg/kg and nivolumab either at 1 or 3 mg/kg. The maintenance dosage of nivolumab of 3 mg/kg every 2 weeks was changed to the equivalent dose of 480 mg every 4 weeks as the study progressed. Bristol Myers Squibb-published guidelines were used to manage immune-related adverse events.

Tumor assessments were done by MRI of hepatic lesions every 8 weeks for 1 year, then every 3 months thereafter. In a minor protocol deviation one patient received external radiation to a sub-diaphragmatic metastasis at month 6, and that lesion was not included as a target lesion. The data safety monitoring committee included physicians uninvolved with the study. Toxicities were graded using CTAE 4.0. Data cutoff was August 30, 2021, with a median duration of follow-up of 30 months.

### Objectives

The primary objective was to determine the safety and tolerability of sequential therapy with ^90^Y resin microspheres and immunotherapy, with the number of doses of immunotherapy received and grade 3–5 toxicities as the endpoints. Secondary objectives were response rates by iRECIST criteria, and progression-free survival (PFS) and overall survival (OS) endpoints. Stable disease was defined as greater than 90 days without progression, and survival parameters were measured from the date of the first ^90^Y infusion.

### Statistical analysis

In this rare tumor the study was initially planned for 18 patients as the minimal number to achieve the primary study objective of demonstrating safety and tolerability. When toxicity necessitated a major change in the dosing of both ^90^Y resin microspheres and immunotherapy, the accrual was increased to 26 patients and the protocol was amended to analyze the first 8 patients as cohort A and the subsequent 18 patients as cohort B. Survival analysis was done using Kaplan–Meier curves, which were compared by the log-rank test. Comparison of groups used *t*-tests with Fisher's exact test, utilizing Prism software.

## Results

Between March 4, 2017 and February 10, 2021, 32 patients were consented and screened. Twenty-six patients were enrolled in the trial, received treatment, and were evaluable for toxicity. One patient was not evaluable for efficacy as they refused further therapy on protocol at 8 weeks. Patient characteristics (Table 1) were typical for a group of patients with uveal melanoma metastatic to the liver entered onto a clinical trial. Although patients were eligible if they had had prior therapy for metastatic disease, all patients enrolled were first-line.

**Table 1.** Patient Demographics and Disease Characteristics

Characteristic	Patients (%)
Total	26 (100)
Male	12 (46)
Female	14 (54)
Age, years, median (range)	65 (35–78)
ECOG performance status	
0	21 (81)
1	5 (19)
AJCC stage (8th edition)	
M1a	16 (62)
M1b	9 (35)
M1c	1 (5)
Prior therapy for metastatic disease	0
Extra-hepatic metastases	6 (23)
Liver-only metastases	20 (77)
LDH > ULN	8 (31)
LDH < ULN	18 (69)
Prior radiofrequency ablation to liver	1 (4)

As suspected, hepatic toxicity proved to be dose-limiting, and the hepatic toxicity experienced by the initial patients in this phase 1 trial necessitated major changes in doses for both the immunotherapy and the ^90^Y. Per protocol design, toxicity was evaluated after six patients were treated. Of the first six patients, five received immunotherapy of whom three patients had grade 3 or 4 hepatic toxicity and one patient had grade 4 hemolytic anemia. Due to these toxicities the data safety monitoring committee decided to reduce the dose of ipilimumab from 3 to 1 mg/kg in February 2018.

In addition, the development of hepatic cirrhosis in one patient in November 2018 was a dose-limiting toxicity. Because this patient had very small volume disease with only one 1-centimeter metastasis, the normal liver received approximately the same dose of ^90^Y as the tumor, calculated to be ∼45Gy. The cirrhosis developed 1 year after entry to the study. Liver biopsy showed little inflammation and the cirrhosis was felt principally due to the ^90^Y rather than the immunotherapy. The hepatic toxicity is listed as due to radiation in the following tables, otherwise toxicity listed as due to radiation is toxicity observed before immunotherapy.

We therefore modified the protocol to limit the radiation dose to the normal liver to 35Gy. After modification of the dosage of ^90^Y toxicity improved, with only one grade 3, one grade 2, and eight cases of grade one hepatic toxicity from the ^90^Y in the subsequent 18 patients. With this reduction in dosage of ^90^Y there was a reduction in grade 3–4 aspartate aminotransferase (AST) elevations, from 37% to 4% (Table 2). Of the 26 patients, 5 did not receive any immunotherapy. The causes include two cases of rapid disease progression, one patient refusal, one case of cholecystitis due to yttrium infusion, and one case of persistent grade 2 hepatic toxicity. Although the grades 1 and 2 hepatic toxicity was frequently asymptomatic, other patients with hepatic toxicity had severe persisting symptoms, particularly in the cases with cirrhosis or cholecystitis.

**Table 2.** Hepatic Toxicities After Yttrium-90 (^90^Y) Resin Microspheres

	Toxicity after ^90^Y	
	High-dose ^90^Y* n* = 8	Modified dose ^90^Y* n* = 18
ALT grades 3 + 4	3 (37%)^a^	1 (4%)
AST grades 3 + 4	1 (12%)	0
Bilirubin grades 3 + 4	2 (25%)^a^	0

^a^ Includes patient 002 who developed cirrhosis 1 year after ^90^Y.

Otherwise this is toxicity seen after yttrium infusions and before immunotherapy.

ALT, alanine aminotransferase; AST, aspartate aminotransferase.

The initial dosing of ipilimumab 3 mg/kg with nivolumab 1 mg/kg every 3 weeks for four doses was derived from the approved schedule for cutaneous melanoma in 2016. When this dosage proved excessively toxic, the dose of ipilimumab was changed from 3 to 1 mg/kg for four doses, keeping the dose of nivolumab at 1 mg/kg. With the doses of ipilimumab and nivolumab both at 1 mg/kg, there were only two grade 2 liver toxicities and no grade 3 liver toxicities in nine patients (Table 3). After discussions of the principal investigators and with the DSMB, in 2020 the dose of nivolumab was increased to 3 mg/kg every 3 weeks during the initial immunotherapy phase as that schedule was becoming widely accepted for combination therapy for cutaneous melanoma.

**Table 3.** Hepatic Toxicity After Immunotherapy by Dose Schedule of Immunotherapy

Immunotherapy dosing	No. patients	Grade 0	Grade 1	Grade 2	Grade 3	Grade 4	Percent grade 3 + 4
Ipilimumab 3 mg/kg Nivolumab 1 mg/kg	5	0	0	2	2	1	60%
Ipilimumab 1 mg/kg Nivolumab 1 mg/kg	9	4	3	2			0%
Ipilimumab 1 mg/kg Nivolumab 3 mg/kg	7	1	2	3	0	1	14%

Reduction of ipilimumab to 1 mg/kg was associated with acceptable toxicity.

The toxicity from the immunotherapy was reflected in the number of doses of combined ipilimumab/nivolumab doses a patient was able to receive during the initial immunotherapy phase (Table 4). Five patients received the initial ipilimumab3/nivolumab1 schedule and received a mean of 2.4 of the planned four doses. With the ipilimumab1/nivolumab1 schedule, the next nine patients received a mean of 3.78 doses, and the increase was statistically significant (*p* = 0.03). With an increase in dosage to ilimiumab1/nivolumab3, the mean doses received fell to 2.33 (*p* = 0.016 compared to ipilimumab1/nivolumab1 group) although there were only seven patients in this last group.

**Table 4.** Doses of Ipilimumab Received in Initial Phase of Immunotherapy by Planned Dosing of Yttrium-90 (^90^Y), Ipilimumab, and Nivolumab

Ipilimumab/Nivolumab dosing schedule mg/kg for initial 4 doses	3 mg/1 mg* N* = 5	1 mg/1 mg* N* = 9	1 mg/3 mg* N* = 7
High-dose ^90^Y	5	0	0
Modified dose ^90^Y	0	9	7
Mean number of Ipilimumab doses received	2.40	3.78	2.14

Differences in number of ipilimumab treatments received between ipilimumab1/nivolumab1 and either ipi3/nivo1 (*p* = 0.03) or ipi1/nivo3 (*p* = 0.023) are statistically significant by two-tailed *t*-test.

Twenty-one patients received at least one dose of immunotherapy. Non-hepatic immune-related adverse events shown in Table 5 were similar to other series of patients receiving combination immunotherapy with ipilimumab and nivolumab. There was one case each of grade 4 hemolytic anemia, grade 2 pancreatitis, and diabetes mellitus. Three patients developed hypothyroidism, with two cases of grade 2 and one grade 3 diarrhea/colitis, and one grade 3 rash. With the exception of the patient who developed cirrhosis of the liver, which was probably due to the ^90^Y, no patient developed a grade 3 or 4 toxicity or discontinued therapy due to toxicity during the nivolumab maintenance therapy phase of treatment.

**Table 5.** Non-Hepatic Toxicities^a^

	Grade 1–2	Grade 3	Grade 4
Diabetes mellitus			1 (4%)
Diarrhea	3 (12%)	2 (8%)	
Colitis		2 (8%)	
Pancreatitis	1 (4%)		
Hypothyroidism	3 (12%)		
Pain	10 (40%)	2 (8%)	
Fatigue	14 (56%)	2 (8%)	
Nausea	13 (52%)		
Emesis	8 (32%)		
Rash	6 (24%)	1 (4%)	
Pruritis	8 (32%)		
Headache	5 (20%)		
Myalgias	3 (12%)		
Anorexia	7 (28%)		
Neutropenia	3 (12%)		
Thrombocytopenia	2 (8%)		2 (8%)^b^
Anemia	6 (12%)		1 (4%)^b^

^a^ Includes all adverse events that were possibly related to study therapy and either grade 3 or 4 or occurred in 10% or more patients.

^b^ Includes one case of severe autoimmune hemolytic anemia with thrombocytopenia.

By iRECIST criteria, the objective response rate was 20%, with one complete and 4 confirmed partial responses in 25 evaluable patients, and a disease control rate of 68%. As shown in Table 6, there was no significant difference in response rates between cohort A, the initial eight patients who received the initial dosing of ^90^Y and immunotherapy, and cohort B who received reduced doses. The median PFS for all patients was 5.5 months (95% confidence interval [CI]: 1.3–9.7 months) and the median OS in our study was 15.0 months (95% CI: 9.7–20.1 months). The median OS of 12.0 months in cohort A who received the original doses of yttrium and immunotherapy is somewhat shorter than the 15.0 months in cohort B, but the difference in survival curves was not statistically significant.

**Table 6.** Responses in All Patients; Cohorts A, B, and by Yttrium-90 Treatments

	Cohort A* n* = 8	Cohort B* n* = 18	Bilobar* n* = 19	Unilobar* n* = 7	All patients* n* = 26
CR	1 (12%)	0	1 (6%)	0	1 (4%)
PRc	0	4 (24%)	3 (17%)	1 (14%)	4 (16%)
PRu	1 (12%)	1 (6%)	2 (11%)	0	2 (8%)
Stable	2 (25%)	9 (53%)	9 (50%)	2 (29%)	11 (44%)
PD	4 (50%)	3 (18%)	3 (17%)	4 (57%)	7 (28%)
NE	0	1	1	0	1
ORR	12%	24%	22%	14%	20%
DCR	50%	82%	83%	43%	68%

Analysis of responses and toxicity are made separately for the eight patients in Cohort A who received the initial dosing of ^90^Y and ipilimumab at 3 mg/kg and 18 patients in Cohort B who received reduced doses of both yttrium and ipilimumab.

CR, complete response; PRc, confirmed partial response; PRu, unconfirmed partial response; stable, stable disease for 3+ months; PD, progressive disease; NE, not evaluable. ORR, objective response rate or CR+PRc; DCR, disease control rate or CR+PRc+PRu+stable.

Exploratory *post hoc* analysis data are presented comparing those who received sequential bilobar therapy with ^90^Y and those that for various reasons received ^90^Y to only one hepatic lobe. Frequently, patients developed progressive disease quickly in the lobe that appeared to be uninvolved on the baseline MRI, reflecting how often the hepatic metastases are diffuse in this disease. The data show numerically better response rates with bilobar therapy, although the differences were not statistically significant.

## Discussion

To our knowledge, this is the first prospective study of sequential ^90^Y resin microspheres and combination checkpoint inhibitor immunotherapy for hepatic metastases for any type of cancer. Our study demonstrated that sequential therapy with ^90^Y resin microspheres followed by combination checkpoint inhibitor immunotherapy for uveal melanoma with liver metastases is feasible and safe if the radiation dose to the normal liver is limited to 35Gy and the dose of ipilimumab is limited to 1 mg/kg. Our results with a 20% objective response rate, 68% disease control rate, median PFS of 5.5 months, and median OS of 15.0 months are encouraging in this refractory cancer.

A recent meta-analysis^15^ of phase II clinical trials with various agents for metastatic uveal melanoma showed a median PFS of 3.3 months and a median OS of 10.2 months. These data were intended to serve as a benchmark, and our results exceed theirs by a considerable amount. The studies most germane and comparable to ours are the two prospective studies of nivolumab and ipilimumab combination therapy in Spain^16^ and the United States.^17^ In the latter 35-patient trial, Pelster observed a 18% ORR, 5.5-month median PFS, and median OS of 19.1 months, all similar to our study. Although we had a higher proportion of patients with normal LDH and stage M1a, representing good prognostic features, we also had a higher proportion of patients with hepatic-only disease, a poor prognostic feature.

Although we are confident that our reduced dosing of ^90^Y is an improvement over the initial “BSA” dose formulae, we failed to demonstrate an optimal dose schedule for ipilimumab and nivolumab when used in this setting. Although the schedule of ipilimumab 1 mg/kg and nivolumab 1 mg/kg every 3 weeks for four doses had little hepatic toxicity, it is not an approved schedule in use for other cancers. The schedule of ipilimumab 1 mg/kg with nivolumab 3 mg/kg has become a preferred schedule for cutaneous melanoma at many centers but we saw 14% grade 3–4 hepatic toxicity in our study at that dose. The schedule of ipilimumab 1 mg/kg every 6 weeks with nivolumab has been approved for lung cancer and mesothelioma and may be worth studying in the setting of ^90^Y administration.

Limitations of our study include the small sample size, the lack of a control population, and the very small numbers of patients treated at each immunotherapy dose, precluding a final judgement on the optimal dosing of immunotherapy in this setting. Because of regulatory requirements and the limited number of patients we were unable to investigate other radiation dosimetry methods^18,19^ that may hold promise for decreasing toxicity. The bispecific antibody tenbendafusp has recently been shown in a controlled trial^20^ to improve survival in patients with metastatic uveal melanoma who are positive for HLA-A2. Like other immunotherapies it appears to have a modest response rate but a greater effect on patient survival, and we await further studies with this new agent.

In summary, sequential ^90^Y resin microspheres followed by ipilimumab and nivolumab appears to be an active and safe therapy for uveal melanoma with hepatic metastases. As combination immunotherapy is becoming a frequent treatment for this disease, we feel our results justify a controlled trial to demonstrate whether ^90^Y resin microspheres add to the utility of combination immunotherapy for uveal melanoma.
